# Reframing Aging and HIV: Integrating Geriatric Care into Primary HIV Clinics to Close the Gap in Access

**DOI:** 10.1093/geroni/igaf122.2681

**Published:** 2025-12-31

**Authors:** Athina Schmidt, Jennifer Sergile, Irina Vovnoboy, Archana Asundi

**Affiliations:** Boston Medical Center, Boston, Massachusetts, United States; Boston Medical Center, Boston, Massachusetts, United States; Boston University - Chobanian & Avedisian School of Medicine, Boston, Massachusetts, United States; Boston University - Chobanian & Avedisian School of Medicine, Boston, Massachusetts, United States

## Abstract

The BMC HIV-Endurance (HIVE) Clinic implemented a “pop-up” geriatric care model to address the reluctance of people living with HIV (PLWH) to leave their primary care settings for geriatric services. Patients reported concerns about losing trusted providers. While systemic challenges at our clinic required an increase to the geriatric referral age to 70. The integrated HIVE model introduced specialized geriatric expertise directly into primary care settings to improve access and improve healthcare outcomes. Despite proactive referrals that originated from the patient’s primary care provider with specific concerns to address, some patients still required guidance to grasp the rationale behind their referral to a geriatric specialist. Many expressed reluctance, perceiving such care as unnecessary and associating it with an outdated notion of aging, underscoring the need for targeted education on the benefits of specialized geriatric services In this paper presentation session, we describe the implementation of the HIVE Clinic and the strategies used to address barriers experienced by PLWH in accessing geriatric care. These strategies include enhanced communication, improved scheduling, and targeted patient education on aging-related needs to keep them safe and independent at home. Additionally, partnerships with local aging services, including visiting nurse associations (VNA), adult day health programs, and meal services, provided further support to patients. Results demonstrated improved healthcare proxy documentation, addressing polypharmacy concerns, expanded referrals, and strengthened care coordination. The HIVE Clinic’s innovative model successfully improved access to geriatric care, emphasizing the value of familiar care settings and targeted patient education to address gaps in HIV-aging care.

